# Poly(aspartic acid) Biohydrogel as the Base of a New Hybrid Conducting Material

**DOI:** 10.3390/ijms222313165

**Published:** 2021-12-06

**Authors:** Adrián Fontana-Escartín, Guillem Ruano, Fiorella M. Silva, Francesc Estrany, Jordi Puiggalí, Carlos Alemán, Juan Torras

**Affiliations:** 1 Department of Chemical Engineering, EEBE, Universitat Politècnica de Catalunya, C/Eduard Maristany 10-14, Ed. I2, 08019 Barcelona, Spain; adrian.fontana@upc.edu (A.F.-E.); guillem.ruano@upc.edu (G.R.); fsil1011@gmail.com (F.M.S.); francesc.estrany@upc.edu (F.E.); Jordi.Puiggali@upc.edu (J.P.); carlos.aleman@upc.edu (C.A.); 2 Barcelona Research Center for Multiscale Science and Engineering, Universitat Politècnica de Catalunya, Eduard Maristany 10-14, 08019 Barcelona, Spain

**Keywords:** conducting polymer hydrogel, interpenetrated polymer network, poly(aspartic acid)

## Abstract

In the present study, a composite made of conducting polymer, poly(3,4-ethylenedioxythiophene) (PEDOT), and a biodegradable hydrogel of poly(aspartic acid) (PASP) were electrochemically interpenetrated with poly(hydroxymethyl-3,4-ethylenedioxythiophene) (PHMeDOT) to prepare a new interpenetrated polymer network (IPN). Different cross-linker and PEDOT MPs contents, as well as different electropolymerization times, were studied to optimize the structural and electrochemical properties. The properties of the new material, being electrically conductive, biocompatible, bioactive, and biodegradable, make it suitable for possible uses in biomedical applications.

## 1. Introduction

Hydrogels are extensive 3D networks made of hydrophilic polymer chains that can retain a large amount of water. There is a wide variety of starting materials and techniques to prepare a specific use in a wide range of applications [1] such as tissue engineering [2], sensing [3,4] and biomedical applications [5]. Recently, the modification of biodegradable hydrogels with conducting polymers (CPs) has become increasingly relevant. The simplicity of the technique and the improvement on the new material properties by partially interchanging their main characteristics, i.e., improving the electrical properties of hydrogels and improving the structural properties of CPs, yield a very interesting class of materials known as conducting polymer hydrogels (CPHs) [6]. Starting on the seminal work of Gilmore et al. [7], many applications have been developed for CPHs such as electrodes in energy storage with high performance [8,9], wearable devices [10], and biomedical and environmental applications [9,11]. Currently, taking into account the difficulty to obtain an intrinsically conductive hydrogel, the new CPHs are mainly designed to conduct electricity by incorporating conducting polymers, metallic particles, and nano-fillers or by introducing either salts or other charged materials that might confer the possibility of ionic conduction through the three-dimensional hydrogel [10].

An increasing interest in introducing CPs in the matrix of hydrogels has been observed over the last decade [12]. Besides the advantages CP might incorporate into CPHs, another potential cause could be because the biocompatibility drawbacks associated with conductive materials, such as metallic nanoparticles or carbon-based materials [13,14,15]. Two techniques, among others, are being predominantly used to confer electrical properties to the hydrogel matrix. On the one hand, in the form of particles and/or nanoparticles dispersed in the reaction mixture used for the hydrogel preparation, this technique is probably the simplest and most straightforward way of CPHs preparation [12]. However, another approach is gaining more relevance based on intrinsic polymerization within the three-dimensional structure of the hydrogel to ensure the formation of interpenetrating polymer networks (IPNs) [16,17,18,19]. Nevertheless, the combination of the two previous techniques has also begun to be explored as a new approach due to the greater ease of the intrinsic polymer for interpenetrating the hydrogel matrix. This is due to the fact that the conductive nanoparticles distributed within the material act as bridges, reinforcing the formation of an internal conductive network [20,21,22].

Poly(aspartic acid) (PASP) is a synthetic polypeptide with a carboxylic acid in the side chain, which has drawn much attention due to its degradability (amide bond) [23,24], acidic properties and/or negative charge, and biocompatibility [25,26,27]. Therefore, PASP-based hydrogels are interesting not only for their biocompatibility and biodegradability but also for their ease of chemical modification thanks to the highly reactive imide ring that contains the poly (succinimide) (PSI) intermediate during the process of synthesis. Thus, PASP hydrogels offer potential advantages over other conventional anionic hydrogels, becoming a promising choice for specific biomedical applications [28]. Despite this, PASP-based hydrogels have not been extensively used for the formulation of conductive composites but rather have been used in a secondary manner, e.g., mixed with silver nanoparticles for its catalytic effect [29] and to incorporate magnetic properties from Fe_3_O_4_ particles [30].

**Figure 1:**
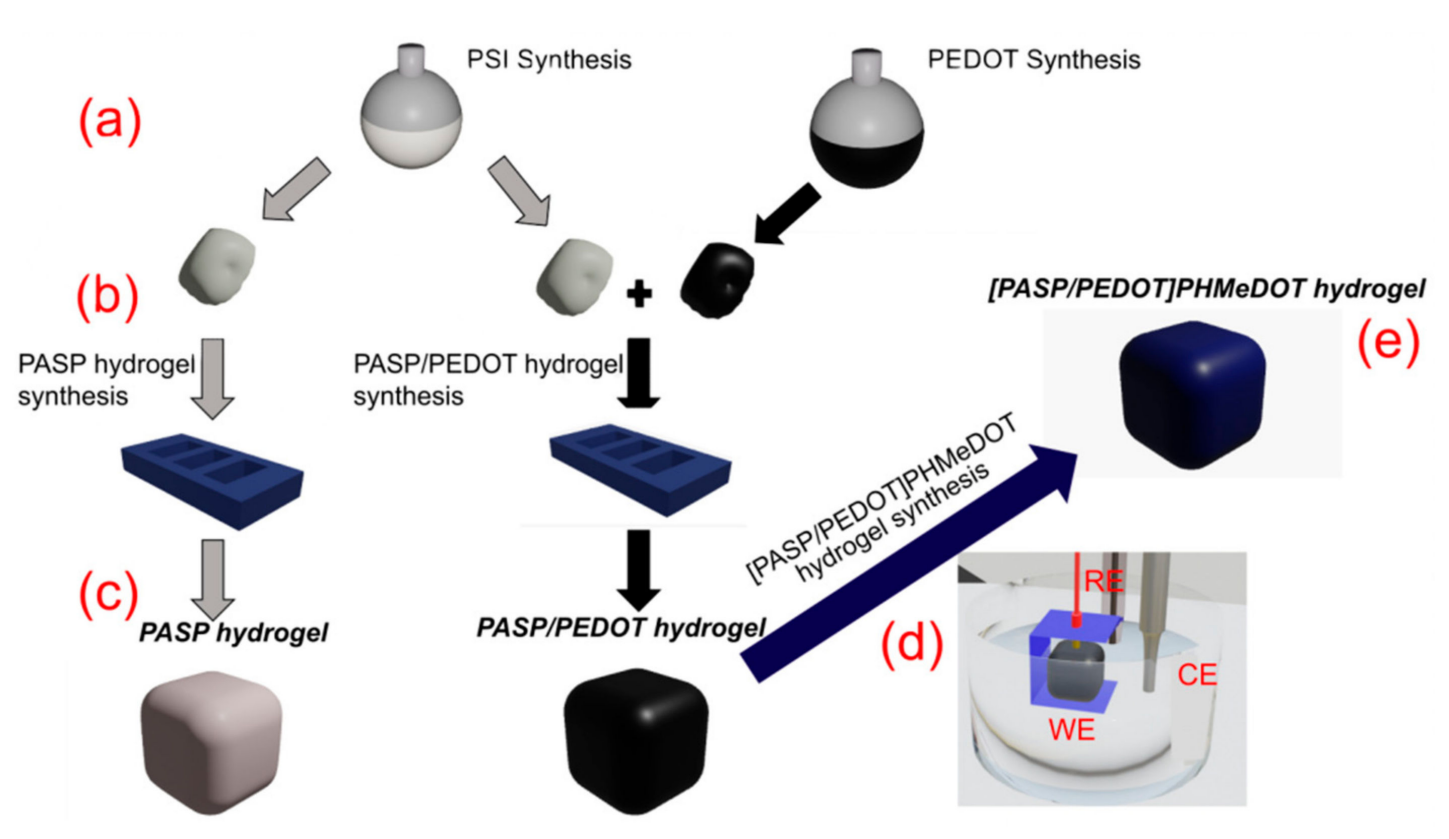
Scheme showing the steps used to produce [PASP/PEDOT]PHMeDOT hydrogels: (**a**) synthesis of poly(succinimide) (PSI) and PEDOT particles; (**b**) the PSI was placed in a mold with and without the addition of PEDOT particles to cross-link and form the different hydrogels; (**c**) PASP and PASP/PEDOT hydrogels were obtained after the gelation stage; (**d**) PASP/PEDOT hydrogel was placed in a HMeDOT solution in order to form a PHMeDOT network by electropolymerization; (**e**) [PASP/PEDOT]PHMeDOT CPHs were obtained.

The aim of this work is focused on obtaining a new PASP-based CPH, in which loaded nanoparticles of PEDOT are interconnected by means of the in situ electropolymerization of poly(hydroxymethyl-3,4-ethylenedioxythiophene) (PHMeDOT) to form a conductive IPN (Figure 1). The new material, hereafter denoted [PASP/PEDOT]PHMeDOT, takes advantage of the main properties of both components, which are the biocompatibility of the PASP and the electro-responsiveness properties of the conductive IPN. As this is the first PASP-based CPH hydrogel reported, up to seven PASP hydrogel formulations were prepared by varying the cross-linking degree of the biopolymer and the amount of loaded PEDOT nanoparticles. These parameters affected the morphology, swelling ratio, and mechanical integrity of the hydrogels, hereafter named [PASP/PEDOT]. The hydrogels with the most appropriate properties were used as electrolytic media for interpenetrating PHMeDOT. The electro-physical and structural properties of the resulting [PASP/PEDOT]PHMeDOT CPHs were evaluated as a function of the time used for the PHMeDOT polymerization, allowing to determine the optimum formulation of the new material.

## 2. Results and Discussion

### 2.1. PSI: Synthesis and Characterization

**Scheme 1:**
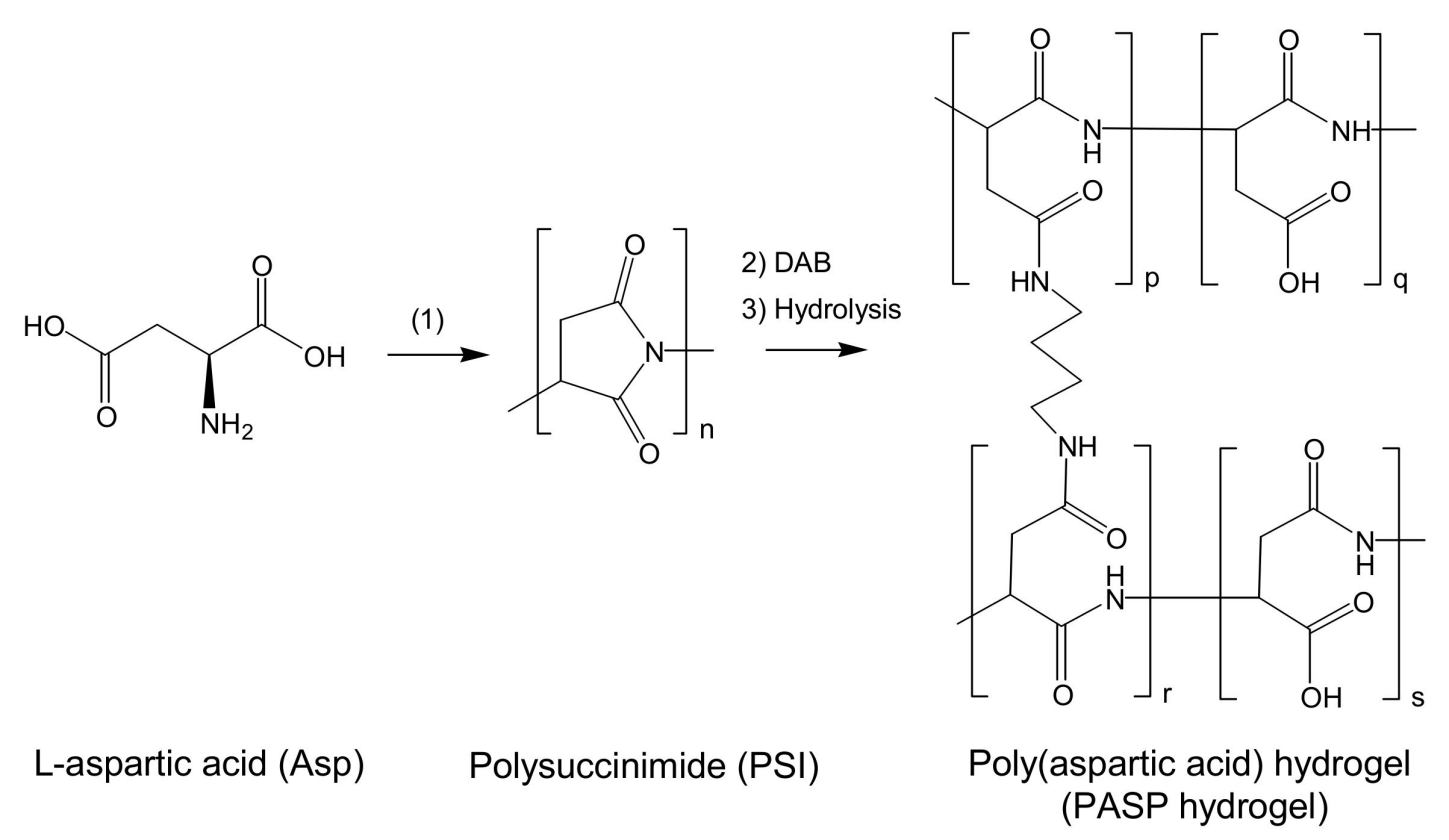
Chemical structure of Asp, PSI and PASP hydrogel and the reaction scheme.

PSI was obtained by polycondensation of L-aspartic acid following the Tomida et al. procedure (see Section 3 and Scheme 1) [31]. After 7 h of reflux using a Dean–Stark trap, as well as its subsequent washing and drying, a yellowish powder was obtained with a final yield of 90.1%. The polymer was characterized by FTIR, ^1^H-NMR, and GPC.

**Figure 2:**
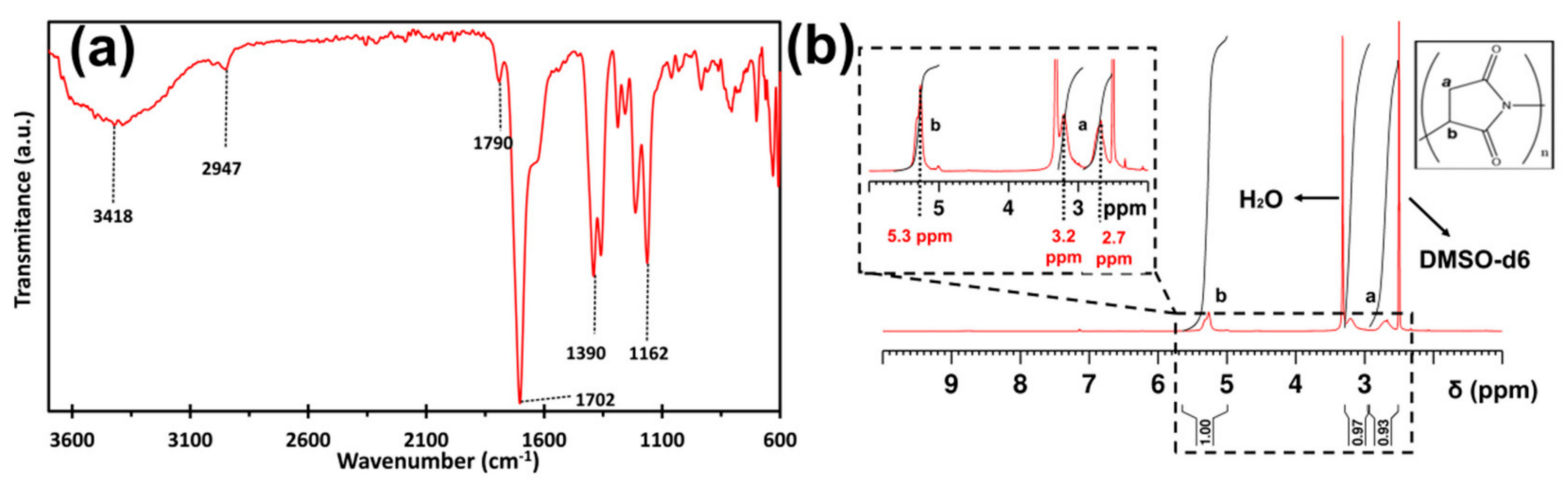
(**a**) FTIR and (**b**) ^1^ H NMR spectrum of PSI in DMSO-*d*_6_.

FTIR spectra of PSI exhibited the following characteristic peaks (Figure 2a): 1162 cm^−1^ (C–C stretching), 1390 cm^−1^ (C–N stretching and N–H bending coupling), 1702 cm^−1^ (C=O stretching), 1790 cm^−1^ (adjacent carbonyl coupling effect due to the ring imide structure), 2947 cm^−1^ (CH_2_ stretching), and 3418 cm^−1^ (N–H stretching) [32,33,34]. The proton NMR spectrum of PSI in DMSO-*d*_6_ is shown in Figure 2b. Signals at 2.7 and 3.2 ppm were assigned to the methylene of the succinimide unit, while the signal at 5.3 ppm was attributed to the methine proton of the same unit [31,35]. Partially superimposed on these peaks can be found the DMSO solvent peak at 2.50 ppm and the residual water peak at about 3.33 ppm due to the hygroscopicity of the solvent [36]. Finally, the elution diagram of PSI by gel permeation chromatography was obtained (Figure S2). The analysis shows a final PSI synthetized weight-average molecular weight (M_w_) of 59,881 ± 1138 g/mol. Moreover, the polydispersity index (M_w_/M_n_) for the PSI is 1.88, with a polymerization degree of 328. These results are in agreement with Tomida et al.’s work [31].

### 2.2. Synthesis of PASP/PEDOT Hydrogel

Initially, different PASP hydrogel samples were prepared by sample criogelation for one week at −18 °C and using the following concentrations of DAB as a cross-linker: 5%, 7%, and 9% of DAB. Hereafter, the resulting hydrogels are denoted PASP:5DAB, PASP:7DAB, and PASP:9DB, respectively. Specially designed molds were built (see Section 3) and used to facilitate the removal of the frozen samples (Figure S1a). The utilization of different concentrations of cross-linker allowed us to optimize the hydrogel formulation in terms of pore size and of both mechanical and structural stabilities for interpenetrating the CP.

**Figure 3:**
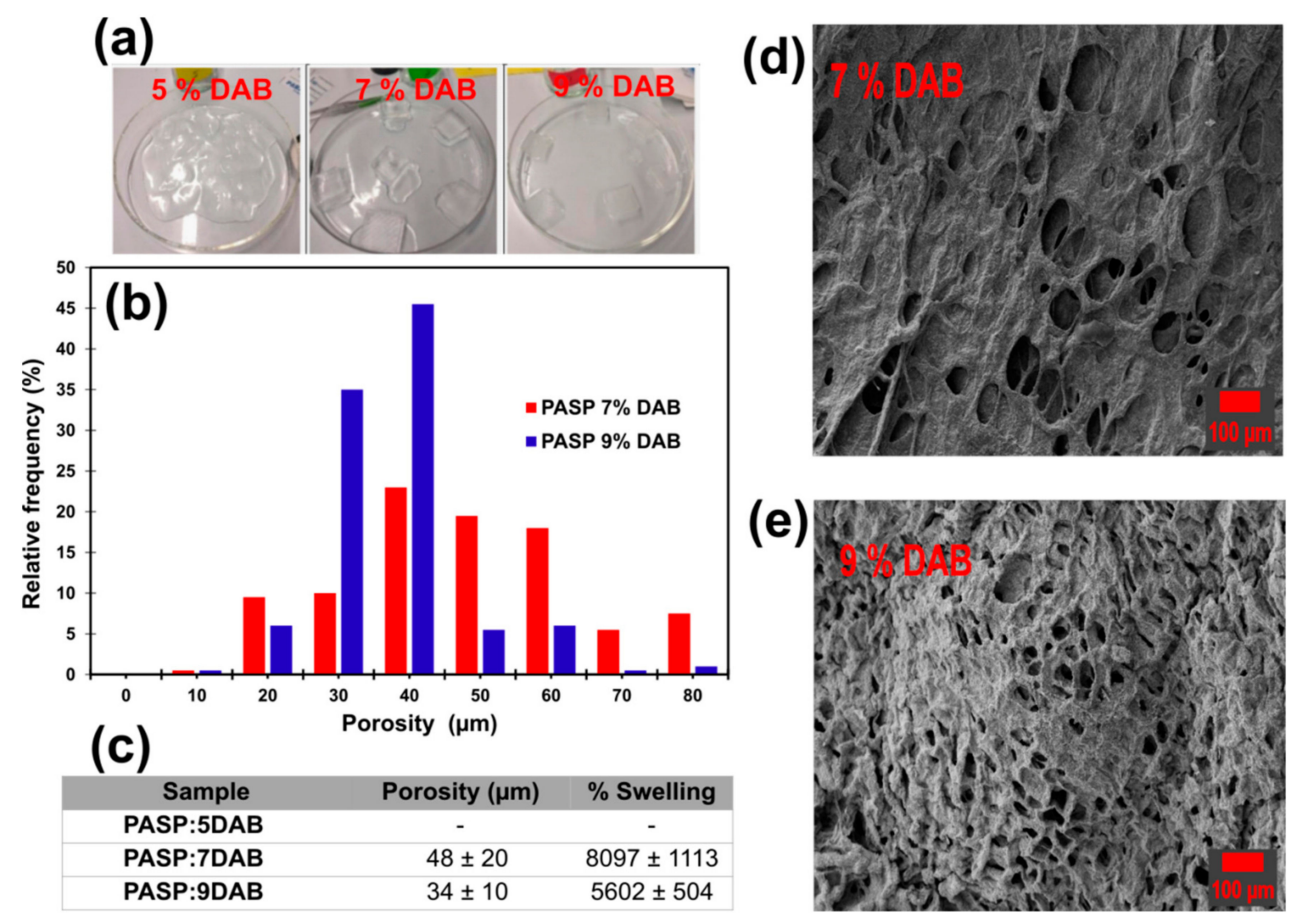
(**a**) Structural stability of cross-linked and swelled PASP hydrogels. (**b**) Pore size distribution histogram of PASP hydrogel synthesized by using different DAB percentages. (**c**) Overview of the porosity and swelling values for the different PASP hydrogels (standard deviation are also shown). Surface SEM images of (**d**) PASP:7DAB and (**e**) PASP:9DAB hydrogels. Micrographs recorded with 100× magnifications are displayed.

The preparation of PASP:5DAB hydrogel was unsuccessful using the employed experimental conditions, as is seen in Figure 3a, whereas the other two DAB formulations were structurally stable. Figure 3b shows the pore size histogram of the PASP:7DAB and PASP:9DAB hydrogels, which were obtained considering 200 measurements on SEM micrographs (250× magnification) of the corresponding freeze-dried hydrogels. PASP:9DAB presented a narrow distribution with an averaged pore size of 34 ± 10 μm, as is shown in Figure 3c. Conversely, the distribution was less homogeneous for PASP:7DAB, which also exhibited a larger pore size (48 ± 20 μm). Consistently, the SEM micrographs displayed in Figure 3d,e reflect that the structure of PASP:7DAB is more heterogeneous than that of PASP:9DAB. The pore sizes obtained in this work are similar to results previously reported for supermacroporous PASP hydrogels synthesized using a similar methodology, even though using different gelation temperatures [37]. Although the average pore size of the former was larger than that of the latter, which could facilitate the subsequent interpenetration of the CP, PASP:7DAB was found to be a very brittle material that easily breaks under simple manipulation. Accordingly, PASP:9DAB was finally chosen for the preparation of the CPHs.

On the other hand, regarding swelling degree, very high values were obtained in both cases, i.e., 8097 ± 1113% and 5602 ± 504% for the PASP:7DAB and PASP:9DAB systems, respectively (Figure 3c). The higher value on the standard deviation of the swelling that presents the PASP:7DAB system is also in agreement with the structural inhomogeneity previously discussed for this hydrogel.

The transformation of supporting materials, such as the γPGA and cellulose hydrogels with poor, or even null, electric properties, into electroactive biomaterials by means of the inclusion of PEDOT micro-particles (MPs), was previously reported by the authors [20,21]. However, in this work, the methodology used to introduce electrochemical properties into the PASP hydrogel was adapted by dispersing PEDOT MPs that were obtained by chemical synthesis. This represents an important improvement with respect to the dispersion of PEDOT MPs obtained by mechanical break of electropolymerized PEDOT films [20,21], as the size and homogeneity of the MPs were much better controlled by chemical synthesis. The average size of chemically synthesized PEDOT MPs, which was determined by dynamic light scattering (DLS), was 0.85 ± 0.02 μm (Figure S3). These were small enough to be trapped within the porosity of the PASP/PEDOT hydrogel but still with enough room to facilitate subsequent interpenetration with PHMeDOT. Moreover, the homogeneity in the size of PEDOT MPs, which was not achieved by the mechanical breaking of the electropolymerized films, allowed us to anticipate a uniform interpenetration process.

**Figure 4:**
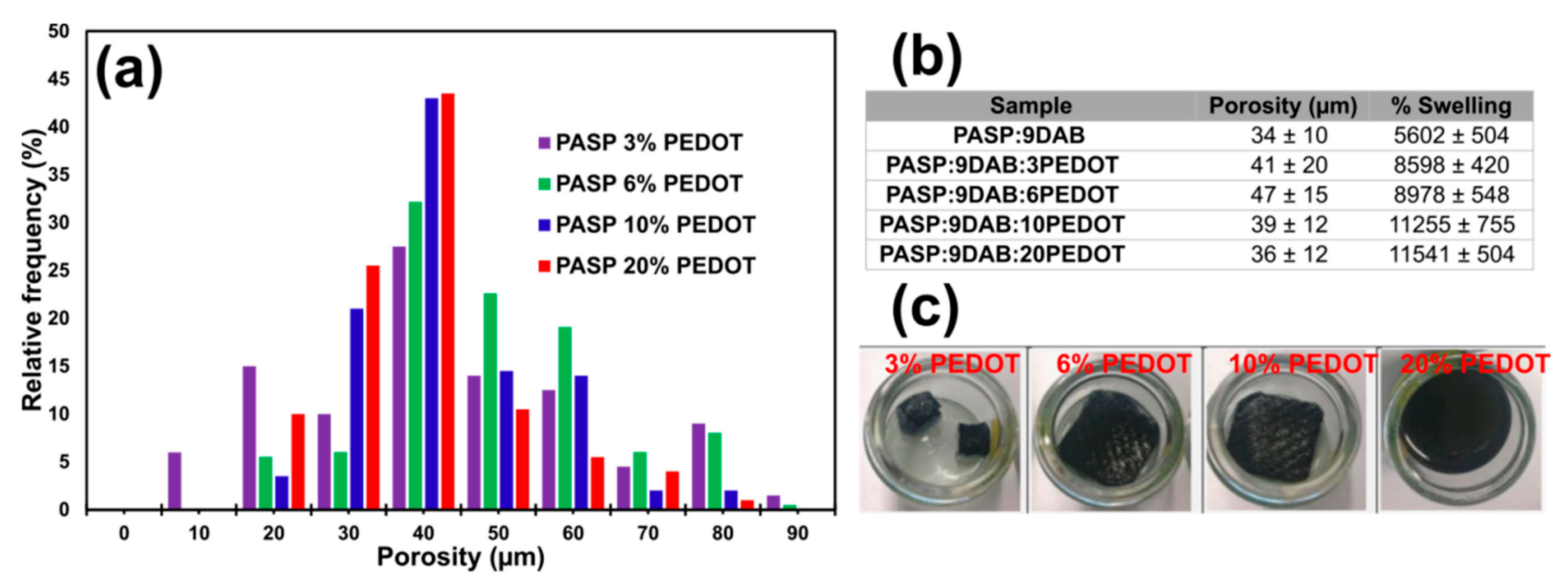
(**a**) Pore size distribution histogram of PASP/PEDOT hydrogels synthesized using different PEDOT percentages. (**b**) Overview of the porosity and swelling values (standard deviation is also shown). (**c**) Structural stability of cross-linked and swelled PASP/PEDOT hydrogels.

The incorporation of PEDOT MPs into the PASP hydrogel matrix was carried out in the step prior to its cryogelation. More specifically, the synthesis of the PASP/PEDOT hydrogel was conducted by dissolving the PSI polymer in DMSO solutions with different PEDOT MPs concentrations (i.e., 4, 6, 10, and 20%) and using a solution of 9% DAB in DMSO as the cross-linker. Next, the mixture was subjected to the gelling process for a week at −18 °C. Figure 4 shows the pore size distribution histogram of the resulting PASP/PEDOT hydrogels. The incorporation of MPs increased porosity as the final percentage of MPs of the hybrid biomaterial grew until a threshold value of 47 ± 15 μm was obtained for 6% of PEDOT. After that maximum, a slow reduction in pore size was observed as the presence of MPs increased up to 20%. Regarding the relative frequency of pore size, a behavior similar to that of the PASP hydrogel with increasing DAB concentration was observed. Thus, as the concentration of conductive MPs increases, the pore distribution is narrower, showing a lower dispersion on the histogram values. However, PEDOT MPs induced a growth in the SR that reached twice the initial values of PASP hydrogel for materials with 10% and 20% of PEDOT (i.e., PASP:9DAB:10PEDOT and PASP:9DAB:20PEDOT hydrogels, respectively). Unfortunately, the PASP:9DAB:20PEDOT system was not structurally stable enough, and it was discarded for the CPH synthesis. Finally, the PASP:9DAB:10PEDOT system was chosen for the preparation of the new interpenetrated hydrogel due to both its pore size and structural stability with the highest content of PEDOT MPs.

**Figure 5:**
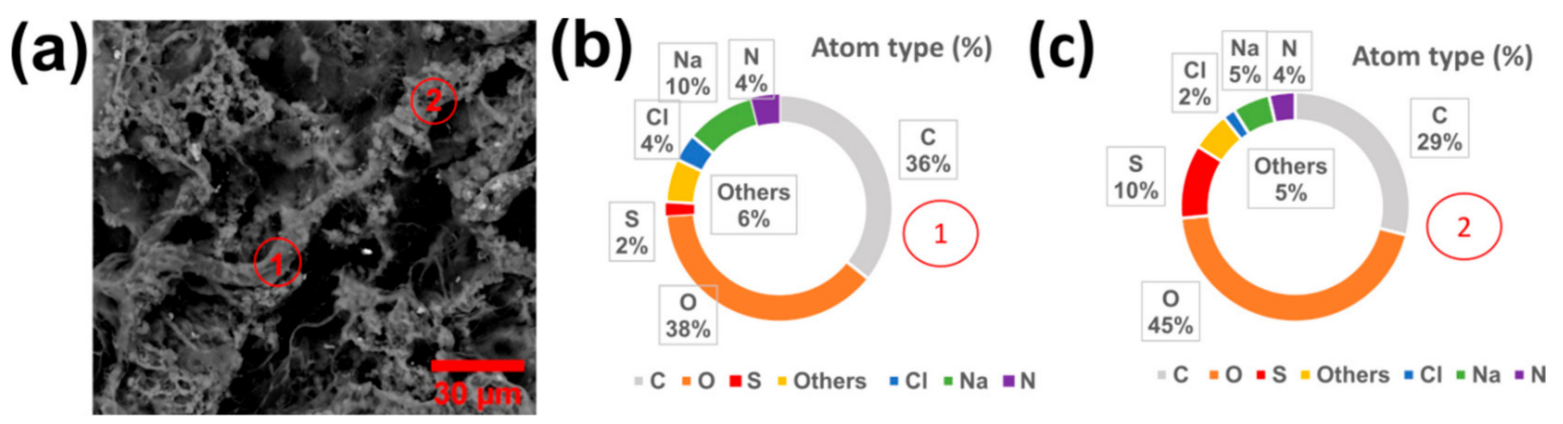
(**a**) Surface SEM micrograph of the PASP/PEDOT hydrogel. EDX analyses determined on (**b**) Point 1 (without PEDOT) and on (**c**) Point 2 (with PEDOT) of the sample displayed in (**a**).

The successful incorporation of PEDOT MPs inside the PASP hydrogel was corroborated by energy-dispersive X-ray (EDX) spectroscopy. Figure 5 shows a representative SEM micrograph of the PASP/PEDOT hydrogel and the results from the EDX analyses on two different surface points (i.e., regions with and without PEDOT MPs within the PASP hydrogel matrix). The S element signal can perfectly be attributed to the thiophene ring of PEDOT, whilst the Cl element is ascribed to the ClO_4_^−^ dopant, allowing an easy differentiation within the surface of the new incorporated PEDOT MPs.

### 2.3. Preparation of [PASP/PEDOT]PHMeDOT Material

A new CHP (i.e., [PASP/PEDOT]PHMeDOT) was created by generating new conduction paths among the dispersed PEDOT MPs within the PASP/PEDOT hydrogel matrix. This was achieved by interpenetrating PHMeDOT doped with ClO_4_^−^ that interconnect the MPs, which acted as polymerization nuclei during the electrogeneration of the CP [20,22]. The formation of such conduction paths was investigated by considering different polymerization times (θ) for PHMeDOT (i.e., θ = 15 min, 30 min, 1 h, 4 h, and 10 h). To facilitate the electropolymerization process of the PASP/PEDOT hydrogel and increase the reproducibility of its subsequent electrochemical characterization, an electrochemical sampler skeleton was built using 3D-printing technology to support the sample and the electrodes of the electropolymerization cell (Figure S1b). The designed structure allowed to expose a constant working area of 0.2 cm^2^ in the sample inside the electrochemical cell, thus increasing its reproducibility and fixing the distances between the electrodes.

The swelling ratio (SR) on the new material induced by applying different electropolymerization times was first determined. Results, which are displayed in Table 1, indicate that the incorporation of PEDOT MPs caused a large increase in the SR of PASP. In addition, the interpenetration of PHMeDOT within the PASP/PEDOT matrix produced an additional increment in the SR. However, this phenomenon, which was smaller than that induced by the incorporation of PEDOT MPs, experienced a slowdown after the first hour of electropolymerization.

**Figure 6:**
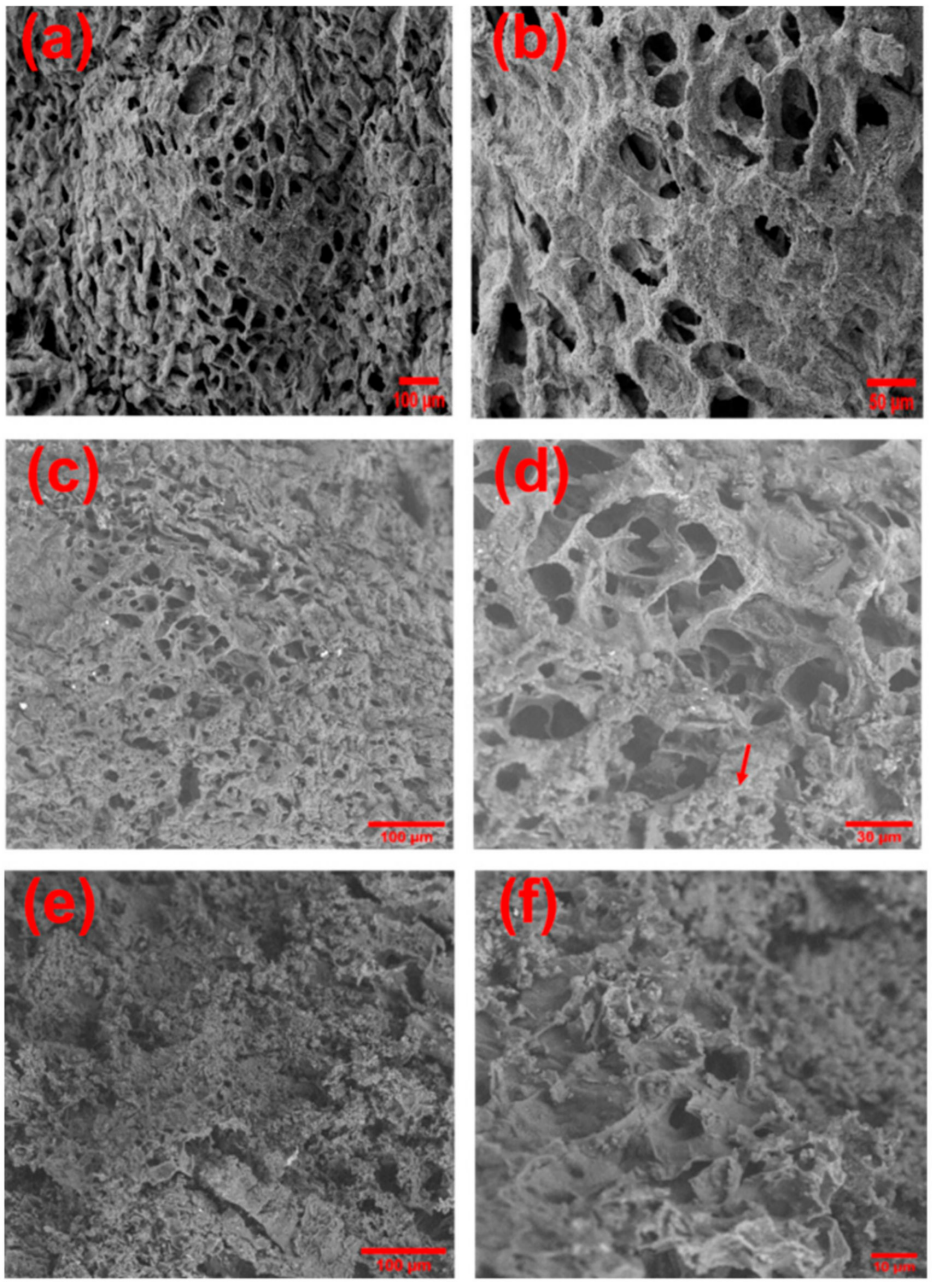
Morphology of freeze-dried hydrogels. Representative SEM images of (**a**,**b**) PASP hydrogel, (**c**,**d**) PASP:9DAB:10PEDOT hydrogel, and (**e**,**f**) [PASP/PEDOT]PHMeDOT (θ = 10 h). Micrographs recorded with (**a**) 100×, (**b**) 250×, (**c**) 450×, (**d**) 1k×, (**e**) 450×, and (**f**) 2k× magnifications are displayed.

SEM micrographs of freeze-dried hybrid hydrogels using two different magnifications, corresponding to a cross-section thereof, are shown in Figure 6. Dense and open macroporous hydrogel structures are observed in all cases. PASP hydrogel with a pore size of 34 ± 10 µm shows a surface with villi due to the residual presence of salt (Figure 6a,b). The inclusion of PEDOT MPs (Figure 6c,d) leads to micropores with a slightly larger mean pore size (39 ± 12 µm) than those shown in Figure 6a,b for the PASP hydrogel. The PEDOT MPs are scattered throughout the interior surface of the pores (indicated by an arrow), which, due to their small size (<1 µm), were identified by EDX analysis (Figure 5). After interpenetrating the PHMeDOT for about 10 h (Figure 6e,f), a much higher amount of CP is observed, which covers the porous surface and differs quite well from the original PASP matrix, showing a rougher surface, not as porous and much closed, with a mean pore size of 24 ± 6 µm.

**Figure 7:**
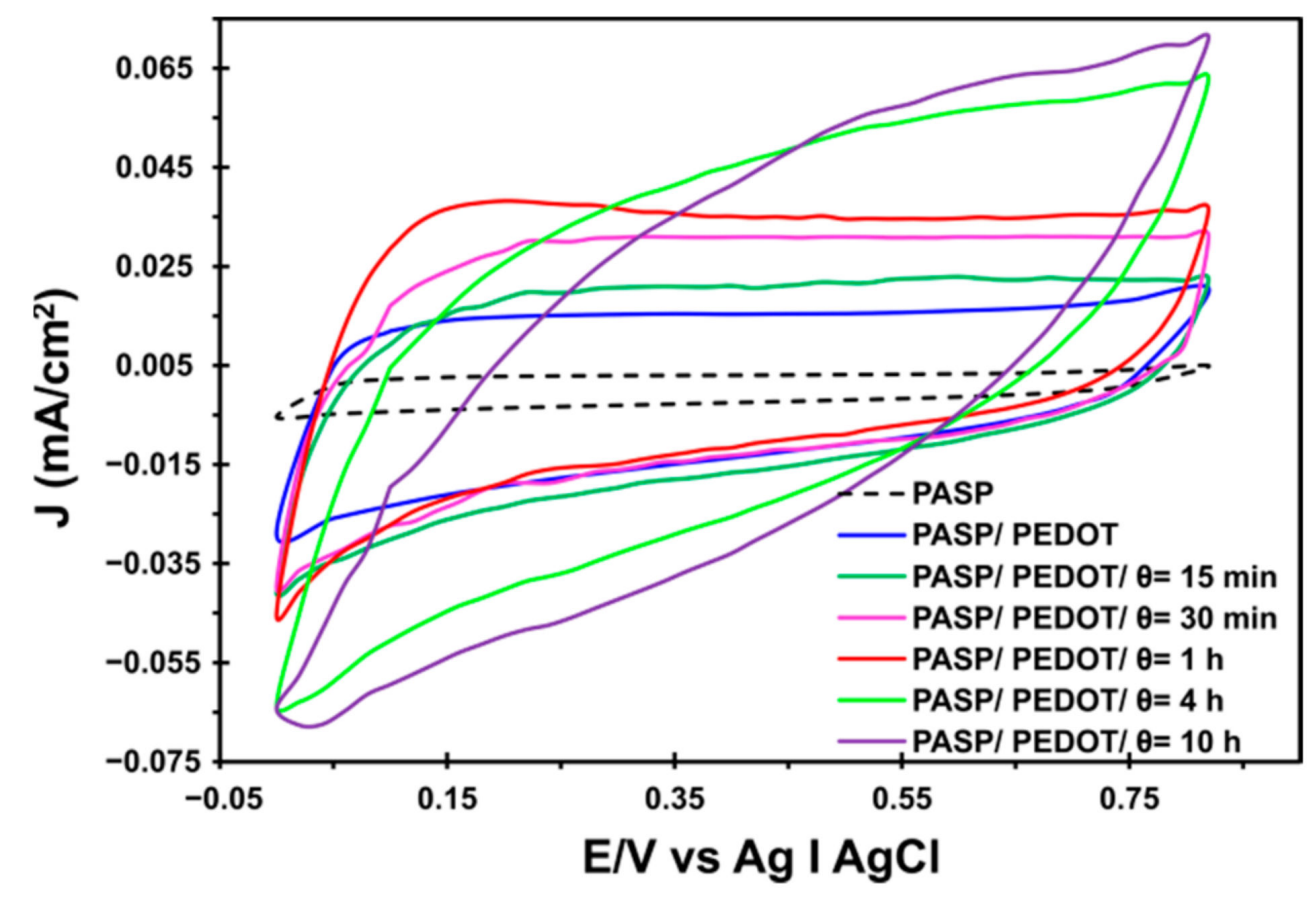
Control voltammograms (second cycle) for PASP/PEDOT hydrogel and [PASP/PEDOT]PHMeDOT hydrogel prepared using different electropolymerization times (θ = 15 min, 30 min, 1 h, 4 h, and 10 h).

Electrochemical characterization by means of cyclic voltammetry allows us to relate the previous morphological study with the electrochemical activity of the generated CPHs. Figure 7 shows in a single graph the voltammogram registered for the second redox cycle of all the studied systems. A significant increase in current intensity was observed when the PEDOT MPs were incorporated, which was attributed to the excellent properties of this CP. Later, the interpenetration of PHMeDOT at different electrogeneration times gradually increased the current density. In addition, it is important to note that a change in the shape of the CVs profiles was appreciated at about θ = 4 h. The voltammograms obtained for CPHs prepared using θ < 4 h exhibit a typical capacitive shape, but after this threshold, the oxidation and reduction peaks appeared more pronounced due to the fact that the amount of PHMeDOT increased significantly. Indeed, the shape of the voltammograms recorded for systems prepared using θ ≥ 4 h resembles the records obtained with supercapacitors prepared with multilayer films of conductive polymers [38]. This suggests that the interpenetration of PHMeDOT is internally restructuring the hydrogel matrix, forming electrically active internal layers, alternating with non-active layers that act as a dielectric, and producing the effect of capacitors in series.

Previously, it has been shown how the hydrophilicity and water retention increase with the content of CP up to saturation at θ = 1 h. However, voltammograms indicate that the system improves the electrochemical properties when the incorporation of PHMeDOT continues (i.e., the current density values and the area of the voltammograms increased to θ = 10 h). Moreover, control voltammograms of [PASP/PEDOT]PHMeDOT at θ = 4 h and 10 h presented the most symmetrical curves and the greatest electroactivity, indicating the presence of an open and porous surface with excellent ionic mobility through the hydrogel/electrolyte interface. Moreover, no preferential zones of oxidation that would bring about changes in the slope of the anodic current were observed.

Table 2 lists the loss of electroactivity (LEA) between the 2nd and 30th cycle, and the specific capacitance (SC) values for the studied CPHs. The incorporation of PEDOT MPs inside the PASP hydrogel matrix was followed by a large enhancement of the electrochemical activity. Indeed, the SC was two orders of magnitude greater for the hybrid PASP/PEDOT system than for the bare PASP hydrogel. However, an important loss of stability was observed in both systems after 30 redox cycles. Indeed, a high LEA value in the bare PASP hydrogel (~42%) was obtained, though even much higher electroactivity loss occurred in the hybrid PASP/PEDOT hydrogel (LEA ~ 62%), as shown in Table 2 and Figure S4. [PASP/PEDOT] PHMeDOT (θ = 10 h) presents a clear drop-off on the CV plot area after 30 oxidation–reduction cycle steps (Figure S4c).

Similarly, the stability of the hydrogel in the early stages of the interpenetration, i.e., [PASP/PEDOT] PHMeDOT θ = 15 min, was also poor. This behavior could be due to the small amount of PHMeDOT initially deposited, which was quickly lost after several consecutive oxidation–reduction cycles. The low stability observed was fixed by applying higher polymerization times, obtaining much better LEA values, and also increasing the SC of the CPH. However, when reaching θ = 10 h, a high instability of the material was observed again (LEA ~ 61%), which could indicate an excessive overload of CP in the hydrogel matrix that would facilitate the detachment of large portions of PHMeDOT and, thus, worsening of the electrochemical activity. This observation is consistent with the previous consideration of the formation of alternate active and inert inner layers. At high electropolymerization times (θ > 4 h), PHMeDOT saturates the internal areas and accumulates in external areas of the hydrogel matrix, forming an initially active asymmetric structure but much less resistant to successive oxidation and reduction cycles. The SC values were consistent with that behavior. In all cases, the SC value decreased after the 30 cycles had elapsed, except in the case of polymerization at θ = 30 min, which experienced a slight increase after some consecutive oxidation–reduction cycles (i.e., from 20.3 × 10^−5^ to 22.9 × 10^−5^ mF cm^−2^ after 30 cycles). This phenomenon was attributed to the small structural rearrangement of the material due to the electrochemical process that favors the interaction between the different chemical components that make up the CPH [20]. Interestingly, a change of three orders of magnitude was observed in the SC values between the bare hydrogel material (PASP) and the last hybrid CPH generated (θ = 10 h), thus confirming the increase in the CPH electroactivity when adding more PHMeDOT to the hydrogel.

## 3. Material and Methods

### 3.1. Materials

l-aspartic acid, phosphoric acid (85%), and hydrochloric acid (HCl, 35%) were obtained from Panreac. 1,4-Diaminobutane (DAB, 99%), imidazole, 4-dodecylbenzenesulfonic acid (DBSA), 3,4-ethilendioxitiophene (EDOT, 97%), hydroxmethyl-3,4-ethylenedioxythiophene (HMeDOT, 95%) were purchased from Sigma-Aldrich (St. Louis, MO, USA). Dimethyl sulfoxide (DMSO, dried, max. 0.025% H_2_O), methanol (MeOH, 99.9%), potassium chloride, mesitylene, and sulfolane were acquired from Merck (Darmstadt, Germany).

### 3.2. Preparation of Poly(succinimide)

Poly(succinimide) (PSI) was obtained by polycondensation of aspartic acid following the Tomida et al. procedure (Scheme 1) [31]. A solution of aspartic acid (25 g, 0.188 mol) and acid catalyst (0.54 mL, 9.4 mmol of phosphoric acid) in 80 g of a solvent (70/30 of mesitylene and sulfolane) was prepared. The reaction was carried at 162 °C under N_2_ atmosphere and left refluxing for 7 h. The water formed in the reaction mixture was removed using a Dean–Stark trap with a reflux condenser. After 7 h, the solvent was removed, and the precipitate was washed one time with MeOH (200 mL) and then three more times with water (200 mL). Finally, the precipitate was washed one more time with MeOH (200 mL) and dried at 85 °C. Once dried, the yellowish powder of the PSI product was left under low pressure to avoid contamination.

### 3.3. Preparation of Poly(aspartic acid) Hydrogels

In order to prepare the PASP hydrogels, the synthesized PSI was cross-linked using 1,4-diaminobutane (in DMSO medium), as shown in Scheme 1. DAB acted as a cross-linker and was chosen among others because no sulfur atoms were present in the molecule, such as cystamine (used in previous works [22]) to facilitate its characterization, thus being discerned from the PEDOT MPs. The solution of the cross-linker (8.8 wt.% DAB in DMSO) was added to the solution of PSI (9.70 wt.% in DMSO). Different cross-linking ratios X_DAB_, defined as the molar ratio between the cross-linker molecules and the repeat units, were attempted (i.e., 0.05, 0.07, and 0.09). After the cross-linker solution was set, the correct amount of DAB was added to the PSI. Then, the new suspension was vigorously stirred and placed in the 3D-printed molds. Finally, these molds were placed in a freezer at −18 °C for 7 days to ensure the gelation of PSI-DAB gel. After the gelation time had passed, the sample-filled molds were immersed into a room temperature aqueous imidazole 0.1 M buffer solution (pH = 8) to yield the final PASP-DAB gels, thus opening the remaining PSI rings. The pH of this buffer solution was adjusted by the addition of 1 M HCl (or 1 M NaOH), and the ionic strength was adjusted to 0.15 M by the addition of KCl.

### 3.4. Synthesis of PEDOT Particles

A total of 1.224 g of DBSA was placed in 40 mL of Mili-Q water and stirred at 40 °C for 1h. Meanwhile, 0.570 g of APS was dissolved in 10 mL of Mili-Q water. After 1 h, 267 μL of EDOT monomer was added to the DBSA solution while keeping it under the previous conditions for 30 min. Then, the APS solution was added to the mix, and the solution was stirred overnight. The resulting PEDOT was washed with acetone and centrifuged three times. Finally, the PEDOT particles were dried in a vacuum medium at 40 °C.

### 3.5. Preparation of PASP/PEDOT Hydrogels

The procedure of preparation of this hydrogel is analogous to the PASP hydrogels. Nevertheless, in this case, the components were readjusted to add the PEDOT particles while still preserving the desired X_DAB_ ratio. Furthermore, four different concentrations of PEDOT particles were evaluated (4, 6, 10, and 20%).

### 3.6. Preparation of [PASP/PEDOT]PHMeDOT Hydrogels

PHMeDOT was electrochemically polymerized inside the PASP/PEDOT hydrogels. This was achieved by submerging the PASP/PEDOT hydrogel into an aqueous solution with 10 mM HMeDOT monomer and 0.1 M LiClO_4_ overnight, which ensured the penetration of the monomer in the hydrogel. Then, using an Autolab potentiostat, a chronoamperometry (CA) was performed at the fixed potential of 0.8 V to initiate the polymerization. The experiments were carried at different polymerization times (θ = 15 min, 30 min, 1 h, 4 h, and 10 h).

### 3.7. Additive Manufacture

Poly(lactic acid) (PLA) filaments were shaped using BCN3D SIGMAX R19 3D printer equipment. Nozzles with 0.3 mm of diameter were used in all cases, whereas the printing temperature was set at 230 °C and the infill density at 20% (sample molds) and 100% (sampler skeleton for electrochemical measures). These devices were designed using SolidWorks software. Regarding the sample molds (Figure S1a), the filaments were shaped as rectangular parallelepipeds of size 19.00 × 5.00 × 0.5 cm^3^ with wells of 1.35 × 3.00 × 0.5 cm^3^. In the case of the electrochemical sampler skeleton, as shown in Figure S1b, a device of size 5.0 × 1.6 × 1.8 cm^3^ was printed with a hole (d = 1.3 cm) to place the electrodes and a cubicle of 1.3 × 1.0 × 1.2 cm^3^ to introduce the samples.

### 3.8. Characterization

The PSI FTIR transmittance spectra were recorded on an FTIR Jasco 4100 spectrophotometer. The powder was deposited on an attenuated total reflection accessory (top plate) with a diamond crystal (Specac model MKII Golden Gate Heated Single Reflection Diamond ATR). For each sample, 64 scans were performed between 4000 and 600 cm^−1^ with a resolution of 4 cm^−1^. In addition, ^1^H-NMR spectra were recorded for the PSI. The experiment was carried out with a Bruker NMR Ascend 400 of 400MHz spectrometer, using DMSO-*d*_6_ as the solvent. For each sample, 64 scans were performed using a sweep at 8 MHz.

Furthermore, the molecular weight of the synthesized PSI was studied through the gel permeation chromatography (GPC) technique. A Waters Alliance 2695 chromatographer was used, with a refraction water 2414 detector. In all cases, a mobile phase of DMF was used (20 mM DMF in LiBr) with Styragel HR4 (7.8 × 300 mm) + Styragel HR4E (7.8 × 300 mm) columns. All experiments were carried out at 35 °C with a flux of 0.7 mL/min.

In order to determine the size of the PEDOT particles, dynamic light scattering (DLS) measurements were performed using a NanoBrook 90Plus Zeta Brookhaven instrument. DMSO was chosen as the solvent because it allows better PEDOT particle stabilization and, thus, minimizes the solvation effect [39] and mimics experimental conditions in the hydrogel gelation process. SEM studies were performed in a focused ion beam Zeiss Neon 40 scanning electron microscope; when using the secondary electron detector, a voltage of 5 kV was used, and a voltage of 10 kV when the measures were performed with the backscattering detector. Furthermore, the microscope was equipped with an EDX spectroscopy system; thus, EDX measures were carried operating at a voltage of 5 kV.

Electrochemical characterization was performed using an Autolab potentiostat controlled by NOVA software. A conventional three-electrode cell was used with the hydrogel as working electrode, Ag|AgCl (KCl, 3 M) as a reference electrode, and a platinum wire as the counter electrode. The electrolyte employed was an imidazole buffer solution (pH 7). Experiments were performed at room temperature, using the cyclic voltammetry (CV) technique, applying a scan rate of 50 mV/s, and a potential interval between 0 and 0.80 V. In all cases, a self-designed 3D-printed sampler was used to perform the measures. All electrochemical measures, swelling experiments, and characterization techniques were conducted in triplicate, and several samples were used for high precision techniques such as SEM or EDX.

## 4. Conclusions

In this study, we report the preparation and characterization of a new CPH based on PASP as a potential biomaterial to be used as flexible electrodes in biocompatible devices. PEDOT MPs were dispersed in a high-porosity PASP hydrogel to confer the necessary electroactivity and, thus, serve as a bridge within the hydrogel matrix for its subsequent interpenetration with PHMeDOT using in situ electropolymerization. Different levels of the interpenetration of PHMeDOT inside the PASP/PEDOT hydrogel were studied. The electrochemical properties of prepared CPHs increase with the electropolymerization time, [PASP/PEDOT] PHMeDOT θ = 4 h being the optimum. The latter exhibits the best electrochemical properties, a high specific capacitance, and a low loss of electroactivity in comparison with the materials obtained using higher electropolymerization times. The latter behavior was explained by an accumulation of PHMeDOT in external areas of the hydrogel matrix at θ > 4 h, with the formation of an initially active asymmetric structure but with much lower electrochemical stability. Furthermore, the improvement of the electrochemical properties was accompanied by a very high swelling ratio that no longer increases as much as the CP content increases. Combining the biocompatibility and mechanical integrity of PASP hydrogels with the electrochemical properties of the interpenetrated matrix of PHMeDOT makes it a promising material for the next generation of biodevices.

## Figures and Tables

**Table 1:** Swelling ratio (SR, %) of PASP, PEDOT/PASP, and [PASP/PEDOT] PHMeDOT hydrogels systems at different anodic polymerization times (θ).

System	Swelling (%)
PASP	5602 ± 504
PASP/PEDOT	11,255 ± 755
[PASP/PEDOT] PHMeDOT θ = 15 min	11,842 ± 438
[PASP/PEDOT] PHMeDOT θ = 30 min	10,659 ± 672
[PASP/PEDOT] PHMeDOT θ = 1 h	13,375 ± 780
[PASP/PEDOT] PHMeDOT θ = 4 h	13,598 ± 526
[PASP/PEDOT] PHMeDOT θ = 10 h	13,915 ± 589

**Table 2:** LEA (at 30th cycle, in %) and SC values (in mF cm^−2^) determined from CV curves at the 2nd and 30th cycles for PASP, PASP/PEDOT, and [PASP/PEDOT]PHMeDOT hydrogels systems at different anodic polymerization times (θ). Standard deviation is also shown.

System	LEA (%)	SC (mF cm^−2^) (×10^−5^)	
	Cycle 30	Cycle 2	Cycle 30
PASP	39 ± 1	0.19 ±0.00	0.12 ± 0.00
PASP/PEDOT	62 ± 1	19.7 ± 0.4	7.49 ± 0.06
[PASP/PEDOT] PHMeDOT θ = 15 min	57 ± 4	16.7 ± 0.9	7.07 ± 0.29
[PASP/PEDOT] PHMeDOT θ = 30 min	13 ± 3	20.3 ± 0.2	22.9 ± 0.3
[PASP/PEDOT] PHMeDOT θ = 1 h	12 ± 3	45.9 ± 1.1	40.3 ± 1.2
[PASP/PEDOT] PHMeDOT θ = 4 h	14 ± 3	141.0 ± 1.6	122.0 ± 3.7
[PASP/PEDOT] PHMeDOT θ = 10 h	61 ± 2	228.0 ± 7.6	88.0 ± 1.8

## Data Availability

The data presented in this study are available on request from the corresponding author.

## Supplementary Materials

The following are available online at https://www.mdpi.com/article/10.3390/ijms222313165/s1.
