# Supplementary material for: Poly(aspartic acid) Biohydrogel as the Base of a New Hybrid Conducting Material
Source: Int J Mol Sci. 2021 Dec 6;22(23):13165. doi: 10.3390/ijms222313165 (PMC8658656; doi:10.3390/ijms222313165)
Supplement: Supplementary file 1 [file ijms-22-13165-s001.zip › ijms-1485271-supplementary.pdf]

Supplementary Information

Poly(Aspartic Acid) Biohydrogels as Hybrid Conducting Materials Based

Adrián Fontana-Escartín,[1,2] Guillem Ruano,[1,2] Fiorella M. Silva,[1] Francesc Estrany,[1,2] Jordi Puiggalí,[1,2] Carlos Alemán,[1,2] and Juan Torras[1,2,*]

[1] *Departament d'Enginyeria Química, EEBE, Universitat Politècnica de Catalunya, C/ Eduard Maristany 10-14, Ed. I2, 08019 Barcelona, Spain*

[2] *Barcelona Research Center for Multiscale Science and Engineering, Universitat Politècnica de Catalunya, Eduard Maristany 10-14, 08019 Barcelona, Spain*

[*] Corresponding author: joan.torras@upc.edu

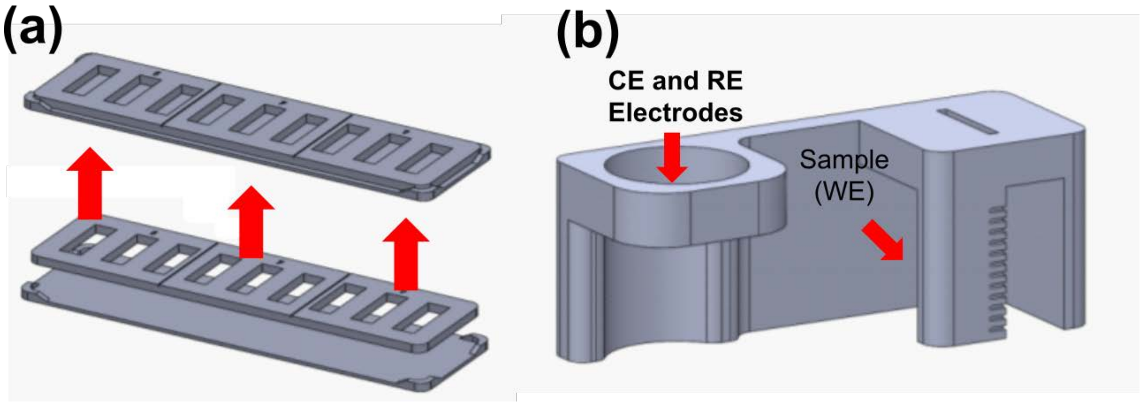

Figure S1. (a) Molds for the synthesis of the PASP and PASP/PEDOT hydrogels ; (b) Sample support device used in the electropolymerization of PHMeDOT and its electrochemical characterization.

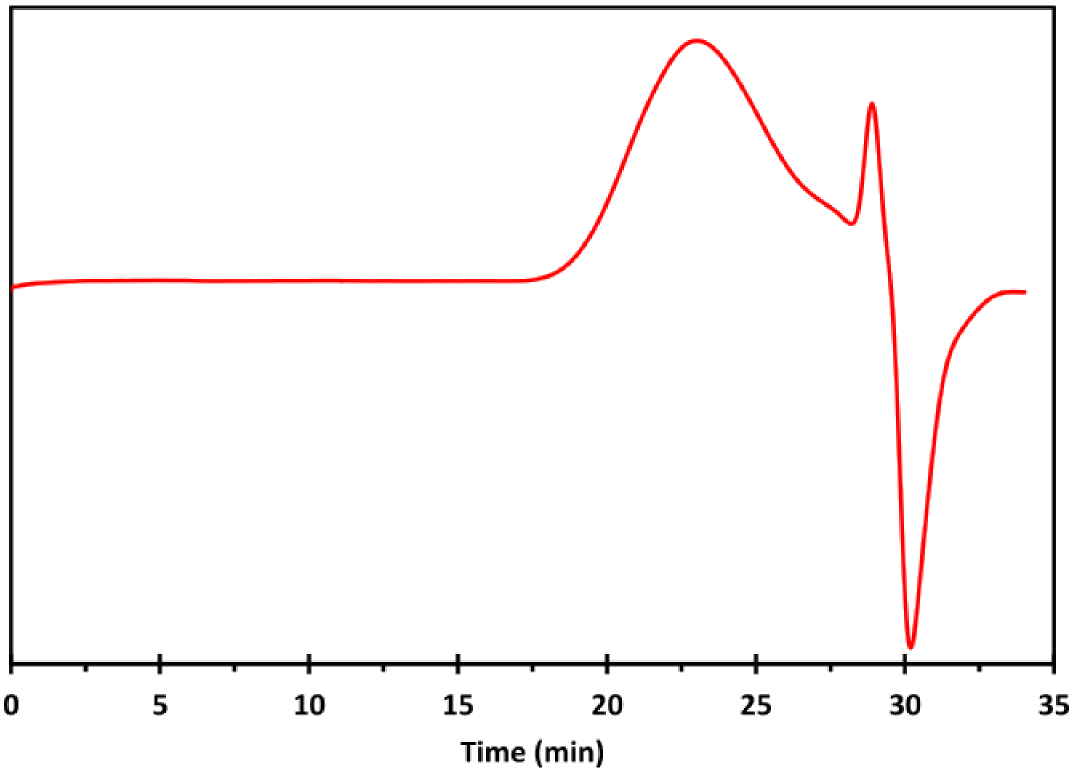

Figure S2. Elution diagram of PSI by gel permeation chromatography (GPC)

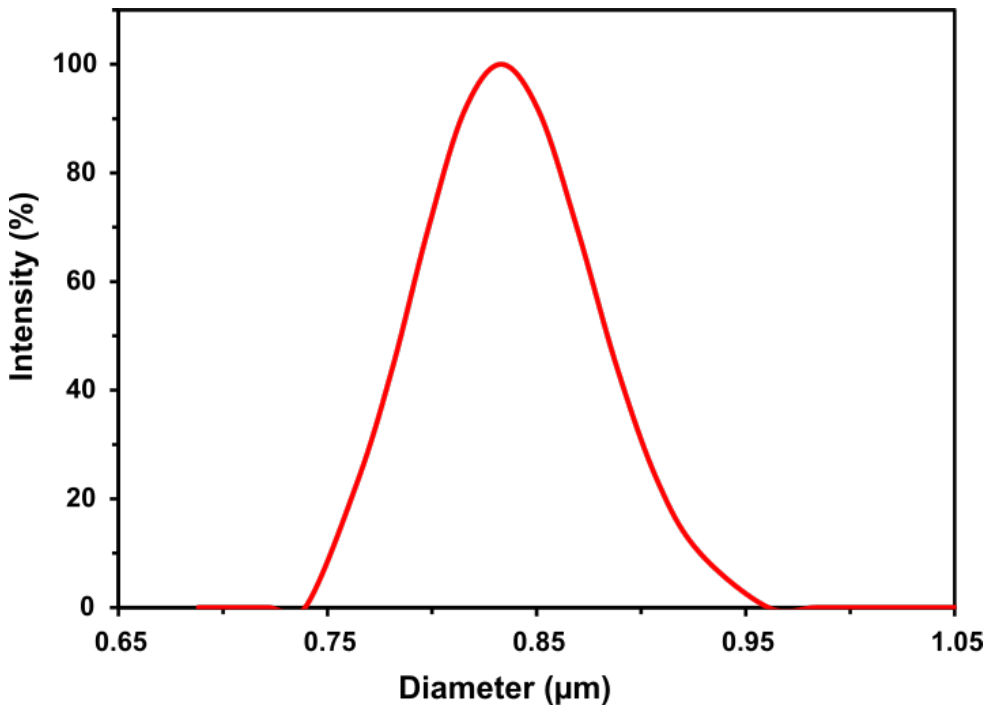

Figure S3. Dynamic light scattering (DLS) spectrum of synthetized PEDOT MPs.

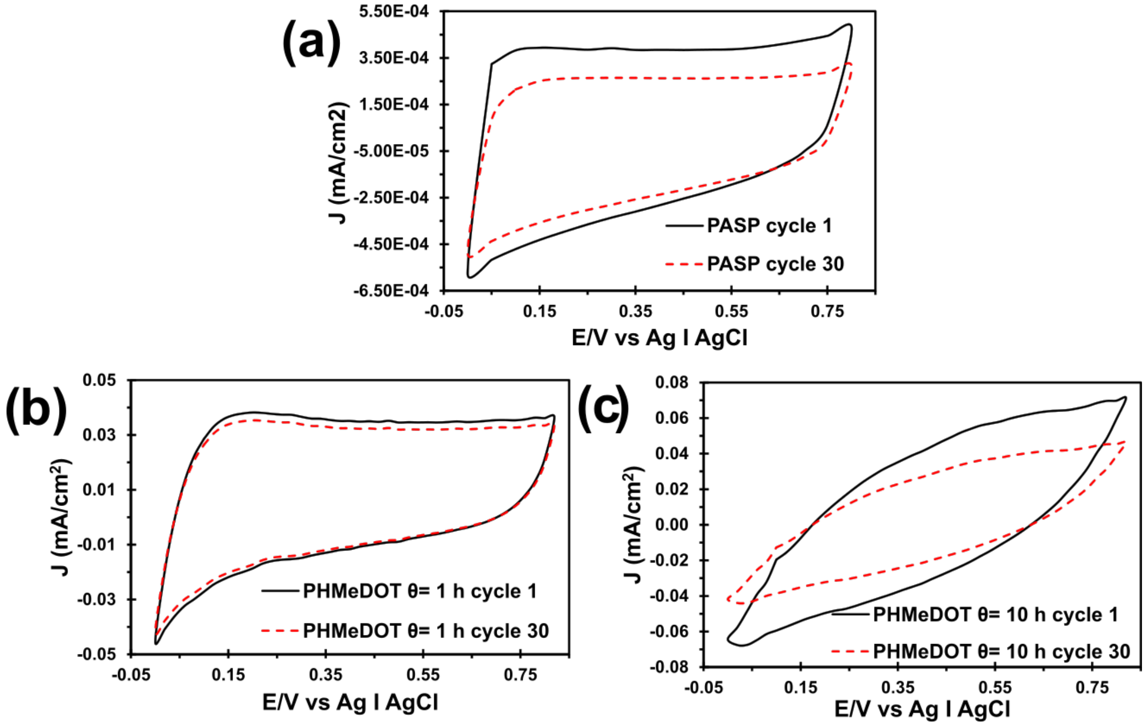

Figure S4. Voltammograms for (a) PASP Hydrogel; (b) [PASP/PEDOT] PHMeDOT (θ = 1h); and (c) [PASP/PEDOT] PHMeDOT (θ = 10h). Both the control voltammogram (first redox cycle) and the voltammogram after 30 consecutive redox cycles are shown.
